# Biomechanical Effects of Incisional Negative Wound Pressure Dressing: An Ex Vivo Model Using Human and Porcine Abdominal Walls

**DOI:** 10.1155/2018/7058461

**Published:** 2018-12-30

**Authors:** Boris Jansen-Winkeln, Stefan Niebisch, Uwe Scheuermann, Ines Gockel, Matthias Mehdorn

**Affiliations:** Department of Visceral, Transplant, Thoracic and Vascular Surgery, University Hospital Leipzig, Leipzig, Germany

## Abstract

**Introduction:**

Incisional negative pressure wound therapy (iNPWT) has been of recent interest in different surgical fields as beneficial outcomes on high-risk wounds have been reported. Nevertheless, its mechanisms of function are not widely studied to date.

**Methods:**

We established two ex vivo setups of iNPWT in porcine and human abdominal wall for measuring pressures within the wound which result from iNPWT application. For pressure measurements, a high-resolution manometry catheter and a balloon catheter probe were used in a wound sealed with either a commercially available PREVENA VAC kit or a self-made iNPWT kit. Furthermore, we evaluated seroma evacuation by iNPWT.

**Results:**

Both setups showed similar characteristics of pressure curves within the wound when applying increasing negative pressures. Application of high pressures did not result in a similar increase in wound pressure. Only subtotal evacuation of seroma by iNPWT application (about 75% of volume) could be detected.

**Conclusion:**

Our ex vivo model of iNPWT in porcine and human abdominal wall could show reproducible measurements of pressures within the wounds in both types of tissue. As intrawound pressures did not increase in the same way as the applied negative pressure, we suggest that our results do not advocate the idea of using iNPWT for wound care especially as seroma evacuation remains insufficient.

## 1. Introduction

Incisional negative pressure wound treatment (iNPWT) has emerged as a useful tool to reduce surgical site infections (SSIs) [1]. Clinical studies showed that, in patients after arthroplasty, iNPWT decreases the rate of wound seroma and inflammation postoperatively [2, 3]. In a silicone wound model, iNPWT could reduce lateral tension and approximate wound edges even in the wound bed [4]. Others demonstrated increased vascularisation along wound edges when using negative pressure [5, 6]. However, very little is known about the specific biomechanical effects of negative pressure on closed incisions/wounds in real tissue.

Therefore, we developed an abdominal wall model to investigate pressure ratios and fluid drainage within closed wounds during negative pressure treatment. Furthermore, we compared a commercially available iNPWT dressing kit with a self-made iNPWT dressing.

## 2. Material and Methods

### 2.1. Abdominal Wall Resectates

A human abdominal wall resectate from a deceased donor (Institute of Anatomy, University of Leipzig, Faculty of Medicine, Leipzig, Germany) was used for the experiments. Procedures with human tissue were approved by the institutional ethics board. Porcine abdominal walls were purchased from a local slaughterhouse.

### 2.2. Experimental Procedures

A standardized wound incision with a depth of 4 cm and a length of 15 cm in the porcine model and 13 cm in the human model was created with a scalpel. Before attachment of the wound dressing, the pig skin was degreased with cleaning solvent (Adler International GmbH, Schwaz, Austria). Wound edges were solely adapted. Clips or sutures were not used.

### 2.3. Negative Wound Pressure Dressings

**Figure 1:**
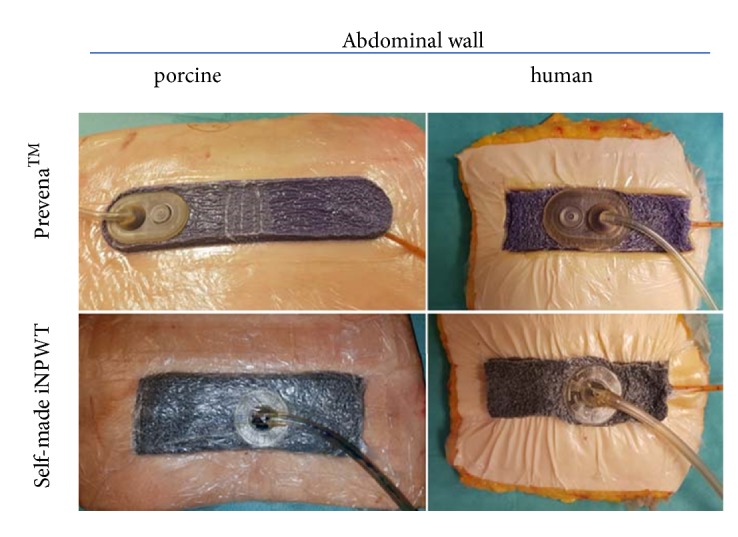
Incisional negative pressure wound therapy (iNPWT) dressings on abdominal wall resectates.

Two different negative wound pressure dressings were tested on both tissues: (1) a commercially available PREVENA™ kit (KCI, San Antonio, USA) and (2) a self-made epicutaneous negative wound pressure dressing made from a regular vacuum-assisted closure (VAC) system (VAC GranuFoam dressing, KCI Medical Products Ltd., Wimborne, UK). The original PREVENA™ dressing (OPD) was applied according to the manufacturer's instructions. Wound edges were overlapped 2 cm on both sides, and the suction was connected via the supplied pad. For the self-made epicutaneous wound dressing (SMD), first the periwound surface was protected with a 2 cm wide plastic foil strip. Then a 4 cm wide black foam, a foil, and suction were attached (Figure 1).

### 2.4. Pressure Measurements

Two different devices were used to measure pressure ratios within the wounds treated with iNPWT: a balloon/hydraulic pressure measuring device and a high-resolution manometry (HRM) catheter.

**Figure 2:**
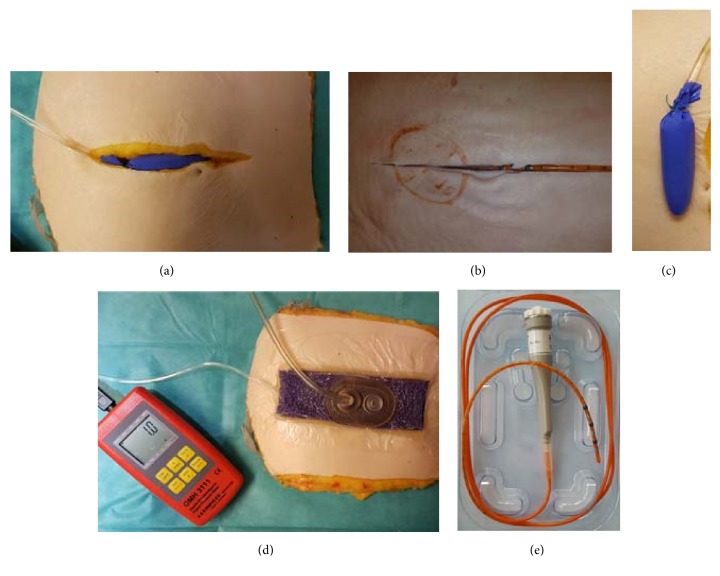
Measurement devices and setup. (a) Balloon in the human model; (b) high-resolution manometry (HRM) catheter in the porcine model; (c) the balloon ligated to the tube; (d) the balloon manometer with original PREVENA dressing (OPD); (e) the HRM catheter.

**Figure 3:**
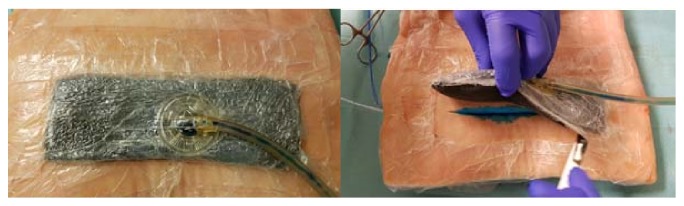
Blue test; (a) the blue indicator fluid appears in the drainage tube; (b) the wound after removal of the dressing.

*We performed four measurement settings*:

(*1) Ballon Pressure with Escalating Suction Power*. To measure wound pressure ratios, a 6 cm long vinyl balloon (made of a disposable glove) was placed around a central infusion line, fixed with a ligature, and placed in the wound (Figures 2(a) and 2(c)). A manometer (GMH3111, Greisinger Electronics, Regenstauf, Germany) and a 10 mL syringe were connected to this catheter through an open 3-way system. After venting 8mL sodium chloride 0.9% solution, the manometer was zeroed. The pressure in this closed system was continuously recorded. After attachment of the wound dressing, escalating suction power was applied (0-50-75-100-125-150-175-200-50-200-0 mmHG) and the pressure values were recorded.

(*2) Pressure over Time*. In the same setting with a new dressing, a continuous negative pressure of 125 mmHg was applied and the pressure was recorded for 20 minutes.

(*3) High-Resolution Manometry with Escalating Suction Power*. A 3-channel high-resolution manometry (HRM) probe (Unisensor, Attikon, Switzerland) for manometry was placed on the bottom of the wound and diverted subcutaneously to the side (Figures 2(b) and 2(e)). The computer-supported recording was performed with the measuring station MMS Solar GI HRM (Laborie Europe, Enschede, Netherlands). Alternating suction levels (0-125-150-175- 200-50-200-50-125-0 mmHG) were applied at 30 sec intervals and corresponding pressure curves were recorded.

(*4) (Seroma) Blue Test. *To simulate formation and evacuation of wound seroma and hematoma at the wound bed, an infusion tube with an inner diameter of 3mm (DI-150 Extension Tube CareFusion, Zibo Ltd., Shandong, China) was placed at the wound base and diverted subcutaneously to the side. After application of the corresponding dressing, a suction of 125 mmHG was applied and methylene blue saline-solution (Patentblau V, Guerbet GmbH, Sulzbach, Germany) was injected at intervals of 60 seconds. As soon as methylene blue appeared in the VAC dressing, the injection was stopped and the VAC dressing was removed after another 30 seconds (Figure 3). The amount of fluid remaining on the bottom of the wound was aspirated and measured. The index between the wound area (length x depth) and remaining fluid volume was calculated to determine the residual fluid in relation to the wound size.

### 2.5. Statistical Analysis

The data were entered in Microsoft Excel 2010 (Microsoft, Redmond, USA) and statistical analyses were performed with SPSS for Windows, release 13.0 (SPSS, Inc., Chicago, USA).

## 3. Results

### 3.1. Balloon Pressure with Escalating Suction Power

**Figure 4:**
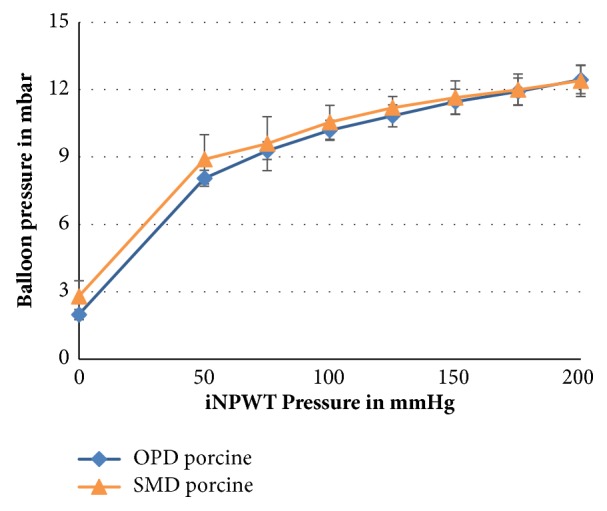
Pressure values in the porcine model using the balloon method (14 measurements with mean value and standard deviation), both the original PREVENA dressing (OPD) and the self-made epicutaneous dressing (SMD). Pressure differences were added to the previous pressure steps.

We compared effects of both iNPWT using the balloon pressure measurement method, on porcine and human abdominal walls in 14 different measurements each (Figure 4). The initial pressure difference (at 0 mmHg iNPWT) is caused by the strength with which the foil was stuck onto the skin. Analysis showed no significant differences between SMD and OPD. Moreover, pressure values in human and porcine tissue were also comparable.

### 3.2. Pressure over Time

**Figure 5:**
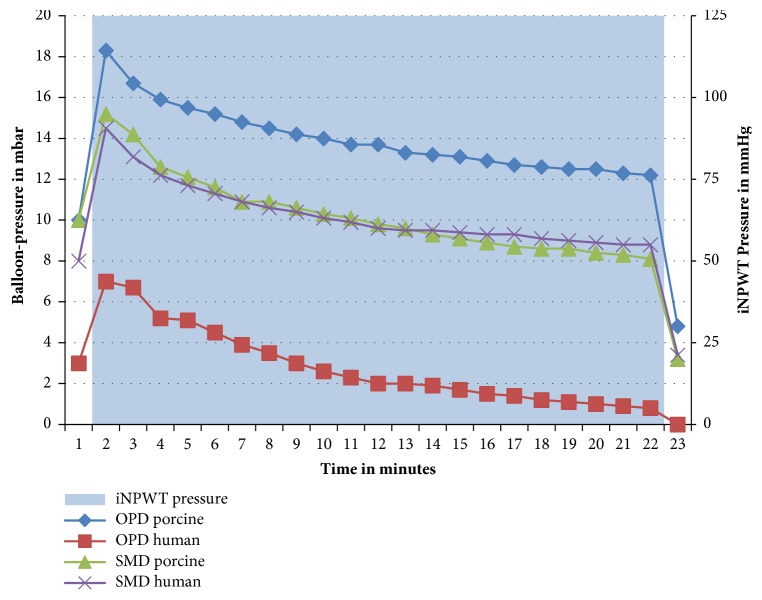
Results of balloon pressure measurements over time in original PREVENA™ dressing (OPD) and self-made epicutaneous wound dressing (SMD), porcine and human. iNPWT: incisional negative pressure wound therapy; OPD: original PREVENA dressing; SMD: self-made epicutaneous wound dressing.

To investigate short-time effects of continuous iNPWT on wound bottom pressure, we measured the “balloon pressure” over a period of 20 minutes with a persistent vacuum suction of 125 mmHg (Figure 5). The pressure values decreased over time, with a median pressure loss of 57% (11 - 67%) after 20 minutes.

### 3.3. High-Resolution Manometry with Escalating Suction Power

**Figure 6:**
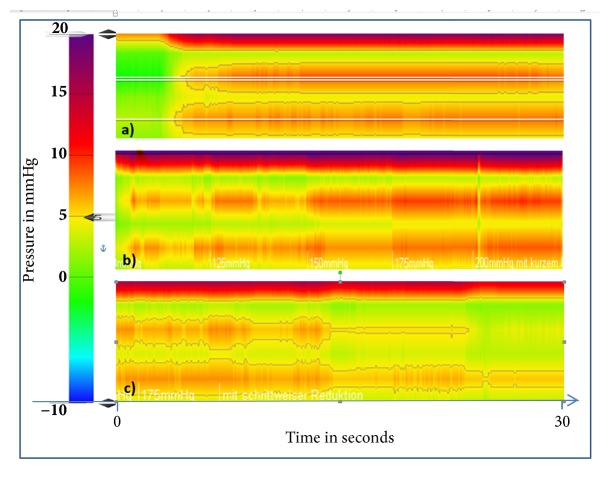
Three examples of high-resolution manometry measurement over 30 seconds: (a) increase of suction from 0 to 200 mmHg; (b) stepwise increase of suction from 0 to 200 mmHg; (c) stepwise reduction of suction from 200 to 0 mmHg. The color coded pressure scale is shown at the left side and ranges from -10 (blue) to 20mmHg (red).

**Figure 7:**
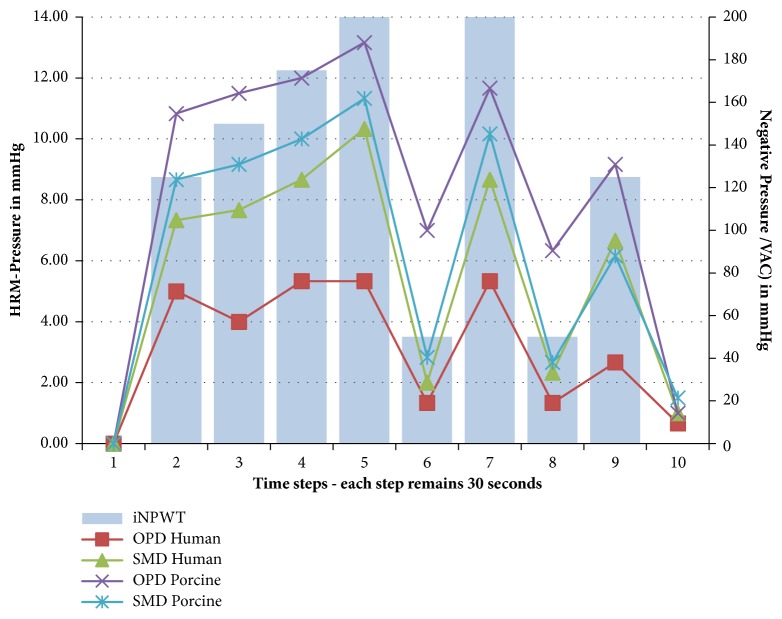
Mean values of the high-resolution manometry (HRM) measurements in the time intervals. HRM pressure analysis left y-axis, incisional negative pressure wound therapy (iNPWT) suction right y-axis. OPD: original PREVENA dressing; SMD: self-made epicutaneous wound dressing.

After applying a vacuum suction, the HRM measurement and the computer-assisted visualization showed a short pressure peak lasting almost one second, followed by leveling at a lower level. The measured values ranged between 0 and 20 mmHg and changed only slightly depending on the vacuum suction applied. Exemplary pressure curves are shown in Figure 6. The means of the measurement values over time are presented with different vacuum intensities in the porcine and human model in OPD and SMD (Figure 7). In accordance with the balloon test method, the measured pressure values are very low.

### 3.4. (Seroma) Blue Test

We performed the blue test to simulate evacuation of hematoma or wound seroma. Up to a threshold volume of 16-18 mL, no release of the seroma via the wound was observed in our experiments. Even after reaching the threshold volume, at which the artificial seroma was released, fluid remained in the wound bed. The amount of remaining fluid ranged between 25 and 31% of the injected volume. The average limit quantity of 17 mL can be compared with the two-dimensional wound surface. This results in an index of 0.28 mL/cm^2^.

## 4. Discussion

In this study, we demonstrated a correlation between external negative pressure applied by the iNPWT system and the pressure within the wound. Even low external negative pressure (about 50 mmHg) led to relevant negative pressure within the wound. Our data is in coherence with previous research of similar physical properties of human and porcine skin grafts [7]. We detected a rather asymptotic increase in wound pressure than a linear increase when augmenting VAC negative pressure. These results suggest approximation of the wound edges through negative pressure wound dressing. In previous studies, Wilkes et al. showed similar effects in 2D computational model without being able to prove it in their silicone model [4]. Surprisingly, we recorded a steady decrease in intrawound pressure over a period of 20 minutes. This might be due to tissue adaptation to our foreign body, i.e., measurement balloon or hematoma, respectively.

Furthermore, we compared effects of a self-made epicutaneous wound dressing (SMD) with a commercial available/original PREVENA™ dressing (OPD). Results of this study are in contrast to our previous work, in which SMD was inferior to OPD in reducing wound infections in a collection of abdominal midline incisions (in press). Lacking factors of live tissue and wound healing (granulation, secretion according to the phase of healing, and movement of the abdominal wall) are possible explanations for this observation.

Seroma evacuation seems to be one of the most important effects of iNPWT [2]. In our blue test seroma model, we revealed a seroma/fluid evacuation after a threshold volume of 16-18 mL. But iNPWT was not able to evacuate seroma in all cases. A remarkable portion of about 25% remained within the wound. This might be insufficient in wounds with high secretion or a large wound. We suggest a wound coefficient of 0.28 mL/cm^2^. In large incisions in obese patients, more than 50 mL of seroma would remain in the wound.

Several works have been performed by Kairinos et al. [8–10] who evaluated suction pressures of NPWT systems placed on superficial and cavity wounds of the extremities. All in vivo NPWT systems were put in place after a thorough wound debridement and a needle probe was inserted directly beneath the dressing or in cavity NPWTs the probe was placed about 1 cm apart from the wound in the adjacent tissues. In the superficial NPWT system, the recorded pressures changed with increasing negative pressure. They reported similar results compared to ours in their superficial NPWT group which resembles the incisional NPWT system: Wound pressure did not increase linearly but rather asymptotically. In contrast to that, the tissue pressure close to the cavity NPWT was not remarkably affected by an increasing negative pressure. They established their NPWT systems on different kinds of sausages to measure pressures at different distances from the wound cavity. They could find a decrease of pressure with increasing distance from the wound cavity. Furthermore their evaluation of long term pressure application showed a steady decrease in wound pressure, similar to our short duration measurement of continuous pressure. All in all, those works seem to be in accordance with our findings in iNPWT. But their wounds differed from ours as they were superficial wounds of the extremities, which do not share the same anatomic properties as abdominal wall incisions. Additionally, NPWT is compared with iNPWT which shows interesting similarities although the technique of application differs remarkably.

In conclusion, sterile dressing and reduction of wound shear stress seem to be the main efforts of iNPWT. It remains unclear if the iNPWT system can effectively drain wound fluids in closed wounds, especially in larger defects. Further investigations are needed.

## Data Availability

The source measurement data used to support the results of this study are available on request from the corresponding author or included in the article.
